# Combination therapy delays antimicrobial resistance after adaptive laboratory evolution of *Staphylococcus aureus*

**DOI:** 10.1128/aac.01483-24

**Published:** 2025-03-14

**Authors:** Maiken Engelbrecht Petersen, Amanda Batoul Khamas, Lars Jørgen Østergaard, Nis Pedersen Jørgensen, Rikke Louise Meyer

**Affiliations:** 1 Interdisciplinary Nanoscience Center, Aarhus University 11291 , Aarhus, Denmark; 2 Department of Infectious Diseases, Aarhus University Hospital 11297 , Aarhus, Denmark; 3 Department of Biology, Aarhus University 683568 , Aarhus, Denmark; The Peter Doherty Institute for Infection and Immunity, Melbourne, Victoria, Australia

**Keywords:** AMR, *Staphylococcus aureus*, antibiotic resistance, adaptive mutations

## Abstract

Antibiotic resistance, driven by misuse and overuse of antibiotics, is one of the greatest threats against human health. The antimicrobial pressure during prolonged antibiotic treatment of chronic bacterial infections selects for resistance. While antibiotic combinations may reduce resistance emergence, antibiotic-tolerant persister cells can serve as a reservoir for resistance development. Therefore, targeting these cells with anti-persister drugs might provide a novel strategy for resistance prevention. In this study, we conducted 42 days of adaptive laboratory evolution using *Staphylococcus aureus* exposed to rifampicin, ciprofloxacin, daptomycin, and vancomycin, alone or in combination with the anti-persister drug mitomycin C. We monitored antibiotic susceptibility daily and assessed phenotypic changes in growth and biofilm formation in evolved strains. Whole-genome sequencing revealed mutations linked to antibiotic resistance and phenotypic shifts. Rifampicin resistance developed within a few days, while ciprofloxacin and daptomycin emerged in approximately 3 weeks. Treatments with vancomycin or mitomycin C resulted in minimal changes in susceptibility. While combination therapy delayed resistance, it did not fully prevent it. Notably, the combination of rifampicin with mitomycin C maintained rifampicin susceptibility throughout the long-term evolution experiment. Sub-inhibitory antibiotic treatments selected for both previously characterized and novel mutations, including unprecedented alterations in the nucleotide excision repair system and azoreductase following mitomycin C exposure. The delayed resistance development observed with combination therapy, particularly mitomycin C’s ability to suppress rifampicin resistance, suggests potential therapeutic applications. Future studies should evaluate the clinical efficacy of anti-persister drugs in preventing resistance across different bacterial pathogens and infection models.

## INTRODUCTION

Antibiotic resistance has been named one of the greatest threats against human health in the coming decades (1), and it is already the second most prevalent cause of death from infections (2). Resistance emerges when bacteria are exposed to antibiotics in various settings, such as hospitals, livestock production, and environments contaminated with pharmaceutical wastewater (3). Some of the most prevalent antibiotic-resistant bacteria in hospital-acquired infections are methicillin-resistant *Staphylococcus aureus* (MRSA) and vancomycin-intermediate *S. aureus* (VISA) (4, 5). The first-line treatment against MRSA infections is vancomycin or daptomycin therapy, and combination therapy with rifampicin is frequently administered if the infection is associated with an implant (6). Resistance to any of these drugs significantly complicates the treatment. Although daptomycin resistance is rare (7), it has been reported (8, 9), especially in VISA and heterogeneous-VISA (10). Intermediate vancomycin resistance is more common (11), and a single study has also reported a daptomycin-resistant, vancomycin-intermediate MRSA isolate (12).

Development of antibiotic resistance is often associated with long-term antibiotic treatment of recalcitrant infections, such as implant-associated infections that involve biofilms. To increase the antimicrobial potency, antibiotic combination therapy is often used to treat this type of infection (6, 13). In theory, combining antibiotics that have different molecular targets also reduces the risk of resistance development, since more mutations are needed to gain resistance (5). For this reason, rifampicin is never used as monotherapy because resistance to this drug emerges easily (6). However, in practice, combination therapy does not always protect against the emergence of resistance (14, 15). The success of this strategy most likely depends on the drug’s mode of action, the number of mutations required for resistance to occur, and the ability of the drugs to act in synergy against the pathogen.

One of the most common mechanisms of antibiotic resistance is modification of the drug’s molecular target, resulting in decreased binding affinity (16). Antibiotics that target a protein are most prone to resistance development, as a single-point mutation may alter the binding affinity. This is the case for rifampicin (17). In contrast, other cellular structures, such as the cell membrane (the target of daptomycin) or the cell wall (the target of vancomycin), require more complex structural alterations to convey resistance through target modification. From this perspective, DNA represents a potentially attractive target for antibiotics. However, DNA is obviously not unique to bacteria, and DNA-targeting antimicrobials will therefore also be cytotoxic. Vice versa, DNA-targeting antineoplastic drugs used in cancer therapy also have antimicrobial activity, and recent research has pointed to mitomycin C as a powerful antimicrobial. Mitomycin C is a chemotherapeutic drug currently approved for treatment of several types of malignant cancers. It has gained increasing attention during the last decade as a potential candidate for drug repurposing to combat recalcitrant bacterial infections (18–21). Unlike most antibiotics, mitomycin C kills bacteria independently on metabolic activity, as it crosslinks DNA leading to cell death (22). This is a favorable trait as it makes mitomycin C effective at eradicating bacterial persister cells (20)—subpopulations of bacteria with high antibiotic tolerance due to their transient inactive state (23). This phenotype is associated with implant-associated infections where bacteria reside in biofilms, out of reach from the immune system (24, 25). Implant-associated infections therefore require months or years of antibiotic treatment if surgical intervention is impossible (26). Due to the lengthy treatment of these infections, persister cells have been linked to the emergence of antibiotic resistance (27).

We hypothesize that using antimicrobials, such as mitomycin C, to kill persister cells will prevent resistant mutants from emerging due to the shorter treatment time. We also hypothesize that mitomycin C is less prone to resistance development because its molecular target (DNA) cannot change its drug-binding affinity through point mutations, and because it has the same antimicrobial activity against all bacterial cells in a population.

The aim of this study was to determine if *S. aureus* can develop resistance against mitomycin C and if combination therapy with rifampicin prevents or delays resistance against this drug as well as other antibiotics. To address these aims, we performed an adaptive laboratory evolution experiment using four clinically relevant antibiotics (ciprofloxacin, vancomycin, daptomycin, and rifampicin) and mitomycin C in monotherapy or as combination therapy with rifampicin. We then identified which genomic mutations occurred during adaptive laboratory evolution to generate resistance and how these mutations affected the general phenotype of the evolved strains.

## RESULTS

### Adaptive laboratory evolution significantly decreases antibiotic susceptibility for all tested antibiotics except mitomycin C

We studied the emergence of antibiotic resistance in *S. aureus* exposed to antibiotics in monotherapy and combination therapy in an adaptive laboratory evolution experiment over 42 days, generating three independently evolved strains for each antibiotic treatment. In adaptive evolution, bacteria are inoculated into a range of antibiotic concentrations, and bacteria from the highest antibiotic concentration that allowed growth are used to inoculate the assay each day, selecting for higher and higher resistance to the drug.

**Fig 1:**
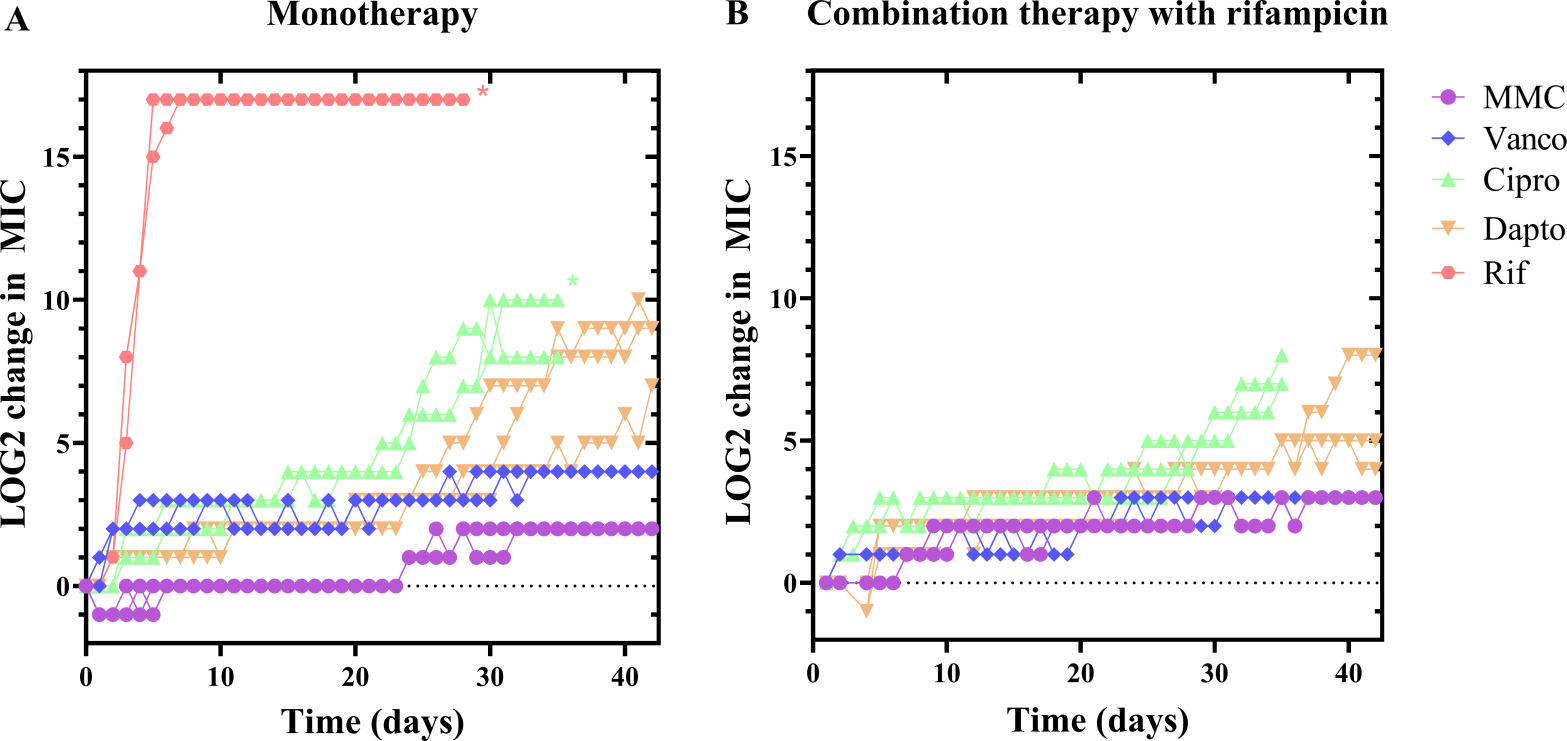
Resistance development during continuous sub-inhibitory antibiotic treatment. *S. aureus* was grown to mid-exponential phase and transferred to 96-well plates containing dilution series of either mitomycin C (MMC), vancomycin (Vanco), ciprofloxacin (Cipro), daptomycin (Dapto), rifampicin (Rif) monotherapy (**A**) or combination therapy (**B**) in combination with rifampicin. The plates were incubated at 37°C overnight, and the MIC was determined by reading plates at 600 nm. Bacteria from wells with antibiotic concentrations immediately below MIC were transferred to a new antibiotic dilution series of the same antibiotic treatment as they had previously received and incubated at 37°C overnight. This cycle was repeated 42 times. As the MIC increased, the concentration range of antibiotics was adjusted to include concentrations from 0.25- to 2-fold the MIC value from the previous cycle. *n* = 3 independent evolutions for each treatment. *The treatment reached the upper limit of detection.

During the 42 cycles of adaptive evolution, MIC values increased for all antibiotic monotherapies. The MIC for rifampicin increased quickly, resulting in >128,000-fold increase after just 7 days (Fig. 1A; Table 1). The increase in MIC for other antibiotics was more gradual, but by the end of the 42 days, MIC values had increased by 4-fold for mitomycin C, 8-fold for vancomycin, up to 128-fold for daptomycin, and up to 1,024-fold for ciprofloxacin (Fig. 1A; Table 1). However, the MIC values for mitomycin C and vancomycin dropped down after making freezer cultures and sub-culturing in an overnight culture, leading to an overall increase in MIC by twofold and fourfold, respectively. The clinical breakpoints for resistance are 0.5 µg/mL for rifampicin, 1 µg/mL for ciprofloxacin, and 2 µg/mL for vancomycin. There are no breakpoint values available for daptomycin and mitomycin C. Breakpoint values are based on several factors: MIC values (measured in Müller Hinton broth) and considerations about pharmacokinetic and pharmacodynamic aspects of the drug (28). The breakpoint/MIC ratios for different pathogens and antibiotics therefore vary from 1 to 250 among susceptible strains (29). Our aim was to investigate the onset of resistance, and to reflect this, we chose to define resistance as occurring when an evolved strain reached a ≥4-fold increase in MIC compared to the parent strain, no matter what the breakpoint value was for the drug of interest. Using this threshold, resistance developed almost immediately for rifampicin, within a week for ciprofloxacin, after approximately 30 days for vancomycin, after approximately 20 days for daptomycin, and never for mitomycin (Table 2).

**TABLE 1:** MIC values of evolved strains^*a*^

	Strain evolved with	Antibiotic tested	MIC of parent strain	MIC post-evolution	MIC post-evolution and culturing	Fold increase (monotherapy relative)	Max. Rif. exposure conc.^*b*^
Monotherapy	MMC	MMC	0.4	1.6	0.8	2	
	Vanco	Vanco	2	16	8	4	
	Cipro	Cipro	1	128, >1,024, >1,024	128, >1024, 1,024	128, >1,024, 1,024	
	Dapto	Dapto	2	1024, 1,024, 256	512, 512, 128	256, 256, 64	
	Rif	Rif	0.008	>1,024	>1,024	>128,000	1,024
Combination therapy	MMC + Rif	MMC	0.2	1.6	0.8	2	0.016
		Rif	0.008	0.016	0.031, 0.063, 0.016	4, 8, 2	
	Vanco + Rif	Vanco	1	8	8	8	0.063
		Rif	0.008	0.063	8, 8, 2	1,024, 1,024, 256	
	Cipro + Rif	Cipro	0.5	64	32	32	0.5, 0.25, 0.25
		Rif	0.008	0.25	4, 4, 2	512, 512, 256	
	Dapto + Rif	Dapto	1	256, 16, 32	256, 16, 16	128, 8, 8	8, 0.25, 1
		Rif	0.008	8, 0.25, 1	512, 1, 2	65,536, 128, 256	

^*a*^ MIC post-evolution was determined from the final cycle of the adaptive laboratory evolution. For combination therapies, pre- and post-evolution MICs were measured in the presence of rifampicin and therefore notes the rifampicin also. MIC “post-evolution and culturing” was determined for single antibiotics after removing antibiotic pressure overnight prior to inoculating the MIC assay. The final “fold increase” in MIC was calculated from MIC values of the parent strains in the absence of rifampicin, and MIC values of evolved strains in the absence of rifampicin. Several values are shown when the three replicate evolved strains did not display the same MIC. In these cases, the numbers are in respective order of strains 1, 2, and 3. All values are in μg/mL.

^*b*^ The maximum concentration of rifampicin that the samples were exposed to during adaptive laboratory evolution.

**TABLE 2:** Days of evolution before resistance emerged^*a*^

	Antibiotic	Days before developing resistance
Monotherapy	Mitomycin C	-
	Vancomycin	33, 31, 29
	Ciprofloxacin	6, 7, 6
	Daptomycin	22, 24, 20
	Rifampicin	3
Combination therapy	Mitomycin C	-
	Vancomycin	-
	Ciprofloxacin	18, 22, 23
	Daptomycin	27, 27, 31

^*a*^ Resistance was defined as reaching a >4-fold increase in MIC. When only one value is written, the number is identical between the three evolved strains. Three values are shown when results from the three replicates differed.

Adaptive evolution was conducted in three independent replicates for each treatment, and resistance developed almost simultaneously in the replicates. The pattern was only heterogeneous in cultures exposed to daptomycin, indicating that changes in daptomycin susceptibility involve several steps. Furthermore, MIC values for daptomycin fluctuated from day to day rather than increasing after each cycle, which could indicate transient antibiotic tolerance rather than resistance (30).

In combination therapy with rifampicin, the MIC for other antibiotics still increased during adaptive evolution, but it generally took longer before resulting in resistance (Fig. 1B; Table 2). For some antibiotics (ciprofloxacin and daptomycin), MIC values at the end of the experiment were lower than for strains evolved under monotherapy (Table 1). However, they were still much higher than the MIC for the parent strain, and we therefore conclude that combination therapy with rifampicin delayed resistance, rather than preventing it.

### Combination therapy with mitomycin C prevented rifampicin resistance during adaptive laboratory evolution and does not cause resistance to other antibiotics

**Fig 2:**
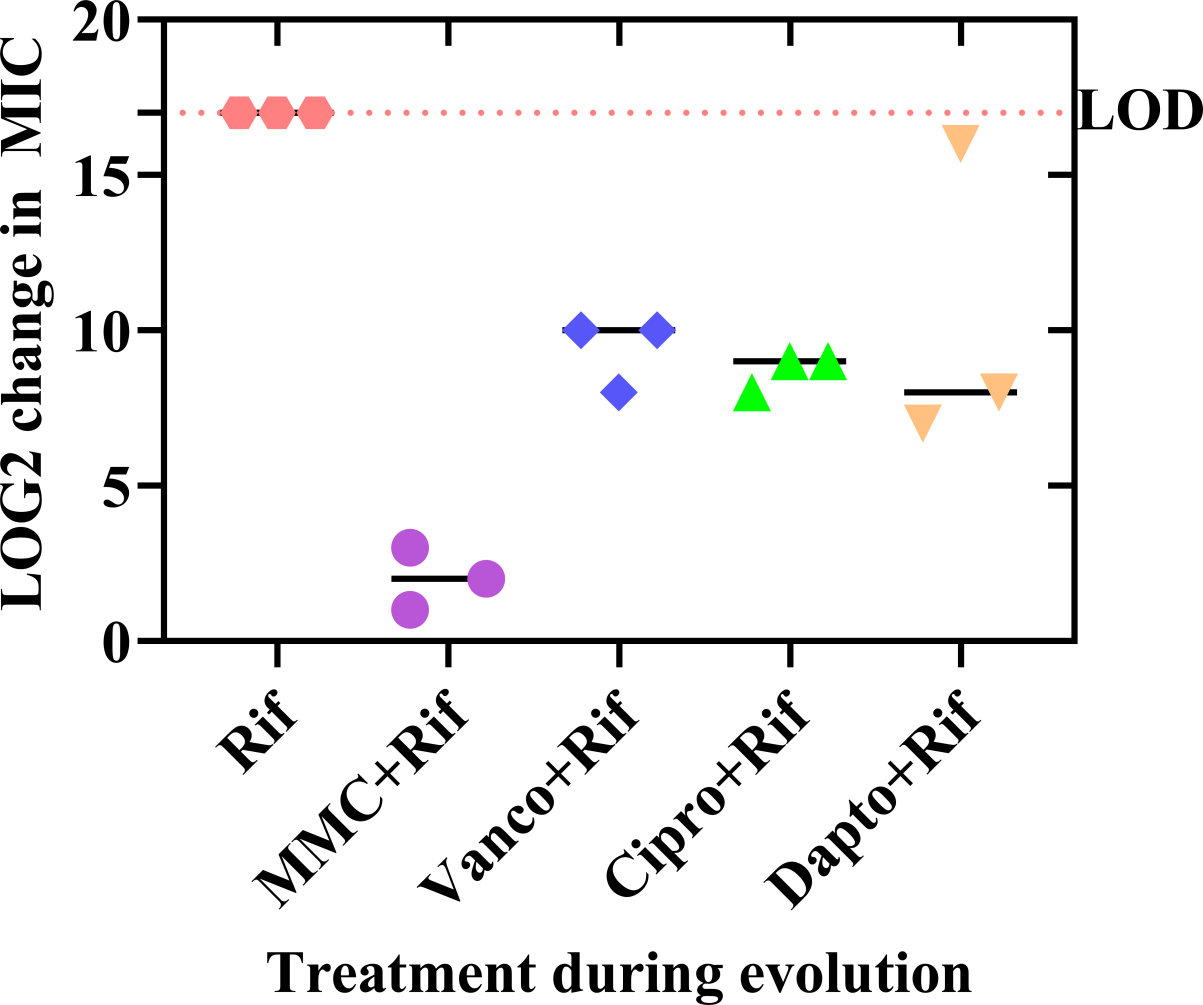
Rifampicin MIC post-evolution of strains receiving combination therapy. Evolved strains were incubated in a rifampicin dilution series in TSB and incubated overnight. The rifampicin MIC was normalized to the MIC of the pre-evolution strain (parent). LOD, limit of detection, the maximum obtainable rifampicin concentration (1,024 µg/mL).

Our primary objective was to determine if rifampicin prevented or delayed the development of resistance to other antibiotics. However, we also measured the MIC of rifampicin after 42 days of combination therapy and found that it was much lower in strains evolved under combination therapy rather than monotherapy (Fig. 2, Table 1). It should be noted that the selective pressure of rifampicin was higher in rifampicin monotherapy compared to combination therapies, and this difference may explain the result (see right-most column in Table 1). However, it is also possible that combination therapy will delay or attenuate rifampicin resistance. Most impressively, the MIC for rifampicin remained below the clinical breakpoint during combination therapy with mitomycin C, and rifampicin MIC only increased 4-, 8-, and 2-fold for the three strains receiving mitomycin C and rifampicin combination treatment, compared with rifampicin monotherapy that rapidly increased MIC >128,000-fold (Table 1). Furthermore, the fold-change from the rifampicin concentration used during evolution to the rifampicin MIC post-evolution was lowest for mitomycin C combination therapy compared to the other combination therapies (Table 1). The mechanism of action of mitomycin C is covalent crosslinking of DNA (22); however, it is not mutagenic (31) and has even been described as inhibiting mutagenesis (32)*,* which may explain the lack of rifampicin resistance development seen here.

In order to determine if the lack of rifampicin resistance in the samples with mitomycin C and rifampicin combination therapy was caused by the rifampicin concentration being too low to exert a selection pressure, we performed a 7-day laboratory evolution experiment. We treated *S. aureus* with rifampicin as monotherapy or as combination therapy with 0.25 × MIC or 0.5 × MIC mitomycin C. We increased the rifampicin concentration as resistance developed, but it did not exceed 0.008 mg/L, which was the highest rifampicin concentration used in the previous adaptive laboratory evolution experiment. After 7 days, all samples were grown at this concentration, and a subsequent MIC assay revealed that even at this concentration, rifampicin was driving rifampicin resistance in all samples (Table 3). Thus, the low concentration of rifampicin used in combination with mitomycin C in the 42-day adaptive laboratory evolution assay is not the cause of the lack of rifampicin resistance, and this effect must be ascribed to the presence of mitomycin C. However, when the “mitomycin C pressure” is eased off by lowering the concentration to 0.5 × MIC, the protective effect is lost.

**TABLE 3:** Rifampicin MICs after 7-day evolution^*a*^

Treatment	Rifampicin MIC post-evolution (µg/mL)
Untreated	0.008
Rifampicin	>128
0.25 × MIC mitomycin C + rifampicin	>128
0.5 × MIC mitomycin C + rifampicin	>128

^*a*^ *S. aureus* was treated with rifampicin alone, with rifampicin + mitomycin C combination treatment or left untreated for 7 days, with daily exchange of media and antibiotics. The rifampicin concentration was increased up to 0.008 µg/mL as resistance developed and maintained at that concentration for the remainder of 7 days. The rifampicin MIC was measured post-evolution after sub-culturing the samples in fresh media.

Prior to performing the adaptive laboratory evolution, we hypothesized that treatment with mitomycin C would cause a large number of mutations due to the DNA-binding nature of the drug. Potentially, these mutations could cause antibiotic resistance to other antimicrobials. During prolonged antimicrobial treatment, it is not uncommon for resistance to arise against other antimicrobials. This has been observed in *Escherichia coli* after evolution using sub-inhibitory concentrations of the chemotherapeutic drug bleomycin, which resembles mitomycin C in its mechanism of action (33). We investigated if evolution under selective pressure from mitomycin C could lead to resistance to other antimicrobials. All three strains that evolved under mitomycin C monotherapy had the same MIC for vancomycin, daptomycin, and ciprofloxacin as the parent strains, and the MIC for rifampicin only increased twofold (Table 4). Therefore, 42 days of exposure to mitomycin C did not lead to mitomycin C resistance or any resistance to other antimicrobials.

**TABLE 4:** MIC values of strains evolved with mitomycin C^*a*^

Antibiotic tested	MIC of parent strain	MIC post-evolution and culturing
Vancomycin	1	1
Ciprofloxacin	1	1
Daptomycin	2	2
Rifampicin	0.008	0.016

^*a*^ To test for cross-resistance, MIC was performed on the three strains receiving mitomycin C during 42 cycles of adaptive evolution and compared with parent strains.

### Evolved strains display phenotypic changes in growth pattern and biofilm formation

**Fig 3:**
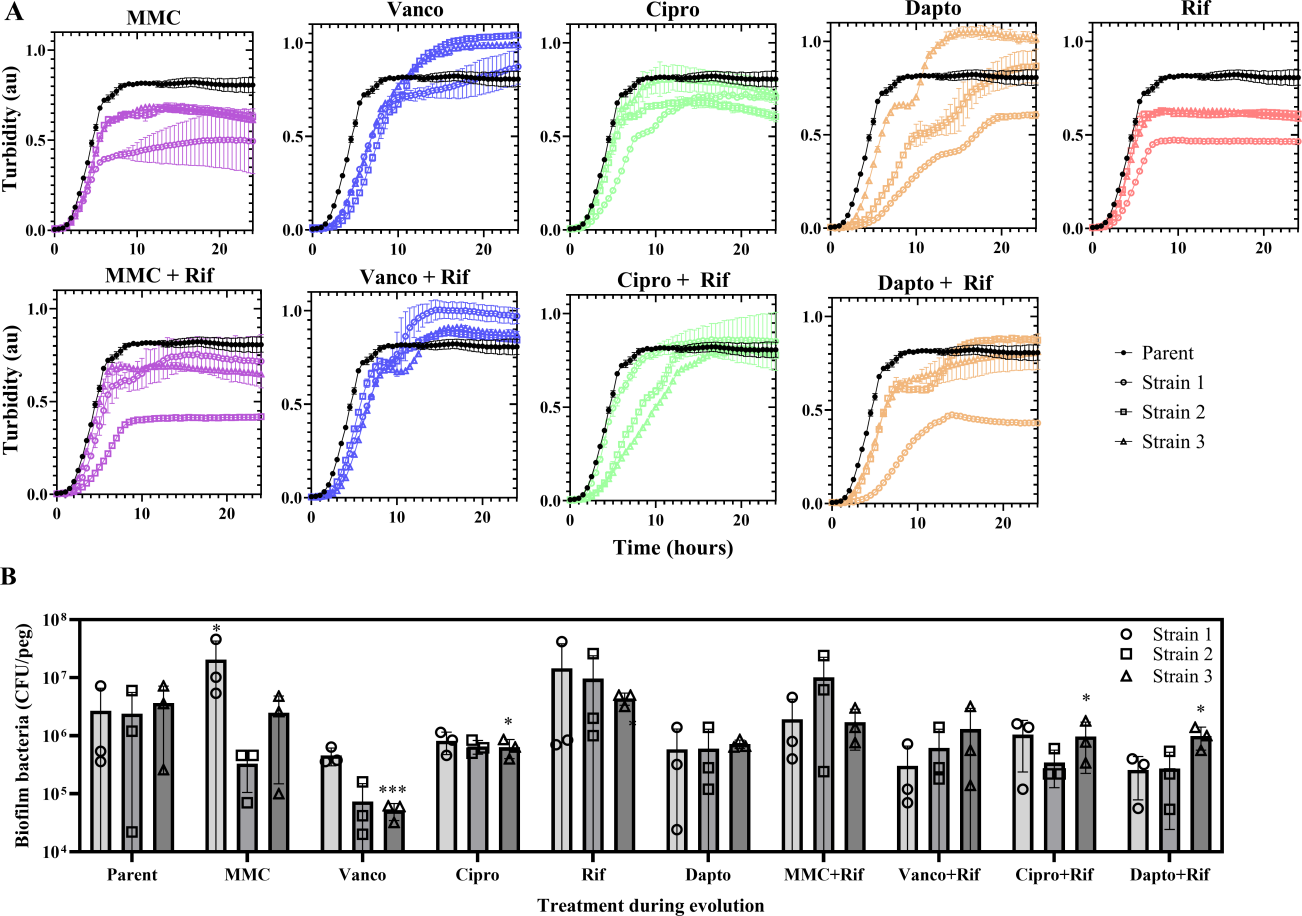
*S. aureus* growth and biofilm formation before and after adaptive evolution. (**A**) *S. aureus* evolved with mitomycin C (MMC), vancomycin (Vanco), ciprofloxacin (Cipro), daptomycin (Dapto), rifampicin (Rif) monotherapy, or in combination therapy with rifampicin were grown in TSB in a 96-well plate at 37°C, and turbidity was measured every 30 min for 24 h. Parent strains are shown in black (mean, *n* = 6, error bars = standard deviation) and evolved strains in colour (*n* = 2). (**B**) Biofilm formed on peg lids in TSB for 3 days media exchange. Viable cells in biofilms were quantified as CFU. Bars show means with standard deviation, and symbols show the individual data points for each replicate sample (*n* = 3 for each independently evolved strain).

Development of antimicrobial resistance is sometimes accompanied by changes in growth patterns that partly explain the ability of the evolved strain to survive exposure to antibiotics. We therefore characterized the planktonic growth rates and ability to form biofilm in all the evolved strains. For all evolved strains, the growth rate either decreased or was unaltered compared to the parent strain (Fig. 3A; Table 5). The evolved strains with the slowest growth rates were MMC + Rif strain 2, Dapto strain 1 and 2, and Dapto + Rif strain 1 (Table 5). Interestingly, the most slow-growing strains evolved under daptomycin exposure (Dapto 1, Dapto 2, and Dapto + Rif 1) also displayed the highest MICs (512, 512, and 128 µg/mL, respectively) relative to the fast-growing strains (Dapto 3, Dapto + Rif 2, and Dapto + Rif 3, with MICs of 128, 8, and 8 µg/mL). Some evolved strains displayed a biphasic growth curve. This phenomenon was most pronounced in strains evolved under daptomycin monotherapy or rifampicin combination therapy with daptomycin or vancomycin. The biphasic growth curve usually indicates a temporary growth arrest after glucose is exhausted from the media (34). Their appearance in evolved strains is indicative of changes in control of metabolic pathways or in the activation of the stringent response, which has been linked to the sudden growth arrest at the point of glucose exhaustion from a complex media (35).

**TABLE 5:** Growth rates for evolved strains and parent strain^*a*^

	Strain	Growth rate (au)
	Parent	1.50 ± 0.03
Monotherapy	Mitomycin C	(1.32, 1.32), (1.40, 1.40), (1.39, 1.39)
	Vancomycin	(1.29, 1.30), (1.24, 1.25), (1.32, 1.32)
	Ciprofloxacin	(1.22, 1.22), (1.39, 1.41), (1.46, 1.48)
	Daptomycin	(1.08, 1.08), (1.18, 1.18), (1.35, 1.36)
	Rifampicin	(1.27, 1.27), (1.45, 1.44), (1.44, 1.45)
Combination therapy	Mitomycin C	(1.37, 1.33), (1.19, 1.20), (1.46, 1.48)
	Vancomycin	(1.31, 1.31), (1.37, 1.37), (1.29, 1.29)
	Ciprofloxacin	(1.42, 1.41), (1.22, 1.25), (1.21, 1.23)
	Daptomycin	(1.11, 1.10), (1.35, 1.35), (1.38, 1.38)

^*a*^ Two measurements were made for each strain (in parentheses) except for the parent strain where *n* = 6. Growth rates were calculated from growth curves (Fig. 3A).

Next, we investigated the ability of the evolved strains to form biofilm in rich media under static conditions. A few evolved strains produced less biofilm, but there was no trend among strains evolved under the same treatment (Fig. 3B). Only a single strain (MMC strain 1) displayed increased biofilm formation.

### Resistance to rifampicin, ciprofloxacin, vancomycin, and daptomycin was linked to the emergence of well-known mutations

After 42 cycles of adaptive evolution, full genome sequencing was performed on the 27 evolved strains and three parent strains to identify mutations that may have caused the resistant phenotype. We assembled the genomes against a reference genome of the parent strain and performed variant calling to identify single nucleotide polymorphisms (SNPs). We compared SNPs identified in the evolved strains with SNPs in the parent strains to identify mutations that had occurred during adaptive evolution. After identifying the genetic mutations, “key mutated genes” were defined as genes that have previously been known to cause resistance, or genes that were mutated at least two times (in the same strain or in two replicate strains) during evolution under a specific antibiotic pressure.

**Fig 4:**
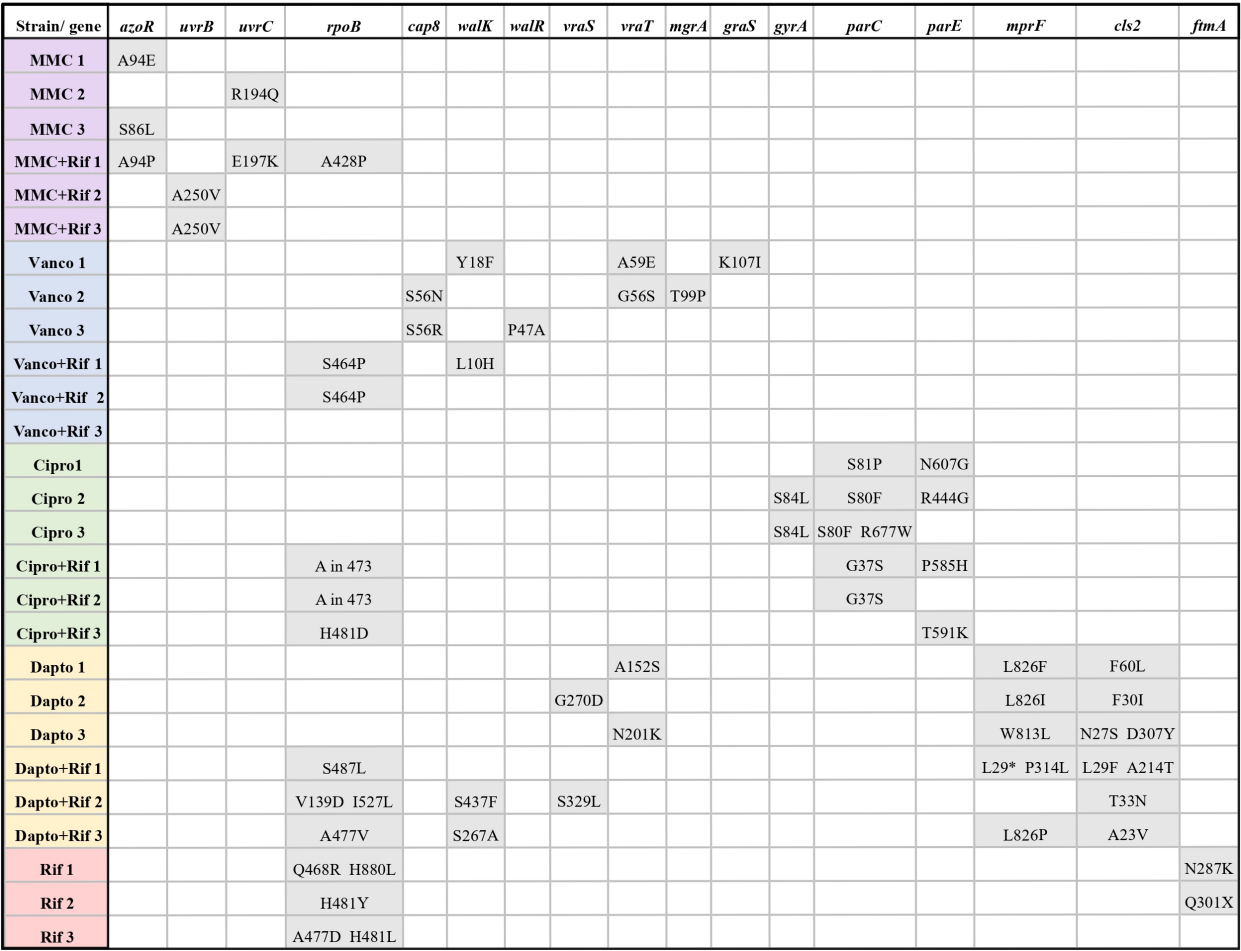
Key mutated genes in evolved strains post-adaptive laboratory evolution. For each gene, the figure shows the amino acid of the parent strain, the amino acid position, and the amino acid after adaptive laboratory evolution.

Rifampicin resistance is well characterized and usually associated with mutations in *rpoB* coding for the RNA polymerase β-subunit (17, 36). We therefore expected to find mutations in *rpoB* in all strains that developed rifampicin resistance. Indeed, mutations in *rpoB* were present in all 12 rifampicin-resistant strains except Vanco + Rif strain 3 (Fig. 4). Surprisingly, a mutation (Ala428Pro) in *rpoB* was also present in MMC + Rif strain 1, which did not display rifampicin resistance. The single amino acid substitution at this position is thus not sufficient to cause resistance. For the two other MMC + Rif strains, we observed no mutations in the *rpoB* consistent with a lack of rifampicin resistance.

Of all evolved strains, we observed the fewest SNPs in strains evolved under rifampicin monotherapy (Fig. S1; Table S2). The mutations were concentrated in *rpoB*, sometimes with more than one mutation in the gene. The only other mutations observed in these strains were in *fmtA*, which encodes the core cell wall stimulon protein, FmtA (37). FmtA is further known to mutate in connection with methicillin resistance in *S. aureus* (38). This finding was surprising, as *fmtA* has not previously been associated with resistance to rifampicin.

As with rifampicin resistance, ciprofloxacin resistance is well characterized and it is often caused by a Ser84Leu mutation in the DNA gyrase subunit A, *gyrA*, resulting in decreased binding affinity of ciprofloxacin to its target (39). We observed this mutation in Cipro strain 2 and Cipro strain 3, which displayed the highest MICs for ciprofloxacin among evolved strains (Table 1). However, these were the only strains with mutations in *gyrA,* and ciprofloxacin resistance in the remaining four resistant strains was most likely caused by other mutations in topoisomerase IV subunits *parC*/*grlA* and *parE*/*grlB*, which are also known to be associated with ciprofloxacin resistance (40–42). Although strains with mutations in only *parC/grlA* and *parE/grlB* showed >4-fold increase in MIC, which we chose as the threshold for resistance, the MIC values were much lower than in strains with mutations in *gyrA*. We further observed mutations in *tilS*, *nrdI*, *gdpP,* and *comEC/rec2*, which have not been connected with ciprofloxacin resistance and which we only observed in single-evolved strains (Table S1). Since these strains also had mutations in genes with known association with ciprofloxacin resistance, we cannot confirm if they are also linked to ciprofloxacin resistance.

Vancomycin resistance was much more complex than rifampicin and ciprofloxacin resistance. We expected to observe mutations in genes that regulate or contribute to cell wall biosynthesis, such as *vraSRT*, *walKR*, *graSR,* and *mprF,* as demonstrated by others (43–47). The three-component system, VraSRT, is a major regulator of the cell wall stress stimulon, which is initiated after *S. aureus* experiences cell wall stress (48). Likewise, WalKR is a master regulator of peptidoglycan metabolism, and it is required for cell viability (49). The two-component system, GraSR, is mostly known for its implications in vancomycin-intermediate resistance (45); however, *S. aureus* relies on it during response to cationic antimicrobial peptides (CAMPs) (50). Likewise, MprF is involved in resistance to CAMPs, as it modifies negatively charged phospholipids and introduces positive charges in the membrane (51). We observed mutations in *graS, walK, walR,* and *vraT* (*yvqF*)*;* however, no particular gene was mutated in all evolved strains, although they reached identical MIC values (Table 1). We further observed mutations in *cap8B* in Vanco strains 2 and 3. *cap8B* is a virulence factor involved in capsule formation (52). Mutations in *cap8B* have not previously been associated with vancomycin resistance in *S. aureus*, but upregulation of *cap8B* has been observed in VISA (53). Vanco strain 2 and Vanco + Rif strain 2 contained mutations in *mgrA*, a global regulator of virulence and autolysis. *mgrA* has been linked to vancomycin resistance in *S. aureus*, but transposon insertion into *mgrA* only increased vancomycin MIC twofold (54) and does not explain the eightfold increase seen here. Therefore, the increase in MIC must be due to multiple mutations in the same strain rather than a single mutation.

Surprisingly, Vanco + Rif 3 showed no mutations in *cap8B*, *mgrA,* or any of the genes associated with cell wall biosynthesis (Fig. 4). Instead, we observed a mutation in *ebh* (Table S1), which is associated with pathogenesis, and mutations in this gene were observed in connection with vancomycin resistance in a single study (43). However, this mutation may have arisen due to the large size (1.1 MDa) of Ebh or mapping errors in one of the 44 repeats (55). We further observed mutations in *uvrB* and *plsY* (Table S1)*,* which have been shown to be mutated in an *S. aureus* isolate from a patient receiving long-term vancomycin treatment (56). Furthermore, we observed a mutation in *ftsW*, which has not previously been associated with vancomycin resistance, but FtsW and vancomycin both bind lipid II without one preventing binding from the other (57). Combined, the mutations in *ebh*, *uvrB*, *plsY,* and possibly *ftsW* may be the cause of the vancomycin-intermediate phenotype in Vanco + Rif strain 3.

A similarly complex picture emerged from the analysis of daptomycin-resistant evolved strains, which contained a multitude of SNPs. We expected mutations in *mprF*, *walKR*, *vraSRT*, and *cls2* due to their importance in cell wall biosynthesis and as they have previously been connected to daptomycin non-susceptibility (8, 58, 59). All daptomycin-resistant strains contained mutations in *mprF* and *cls2*, except Dapto + Rif strain 2 which did not have mutations in *mprF* (Fig. 4). Leucine 826 was mutated in three of the six daptomycin-resistant strains: Dapto strains 1 and 2 (Leu826Phe/Ile), which displayed the highest MIC values (512 µg/mL), and Dapto + Rif strain 3 (Leu826Pro) which had an MIC of (16 µg/mL). Dapto strain 3 had a mutation in a close position (Trp813Leu), and the MIC value was at the >100 µg/mL level (Table 1).

Strains with mutations in the *vraSRT* system were evolved under daptomycin monotherapy and displayed >100 µg/mL MIC values. Dapto strain 2 and Dapto + Rif strain 2 had a mutation in *vraS*, while Dapto strains 1 and 3 had mutations in *vraT*. Mutations in *vraT* are known to cause resistance to vancomycin (44) with observations of *vraT* in vancomycin-intermediate, dalbavancin non-susceptible *S. aureus* (60). However, there is a high occurrence of cross-resistance between vancomycin and daptomycin with a linear correlation between the two antibiotics’ MICs (61). Indeed, we observed mutations in the *vraSRT* in both daptomycin- and vancomycin-resistant strains. Like *vraSRT*, we also found mutations in the *walKR* system among both daptomycin-resistant and vancomycin-intermediate strains, as Dapto + Rif strains 2 and 3 had mutations in *walK*.

### Evolution under mitomycin C pressure induces mutations in *azo1* and *uvrB/C*

We observed a modest (two- to fourfold) increase in the MIC for mitomycin C during evolution under mitomycin exposure as mono- or combination therapy. Although the increase was modest, the change was consistent in all six evolved strains. Mutations that affect bacteria’s susceptibility to mitomycin C have previously been reported in *E. coli*, but only in connection with decreased, not increased, MIC. The gene identified to affect mitomycin C susceptibility was *uvrB* of the nucleotide excision repair (NER) (62, 63). We identified SNPs in the NER-system *uvrABC* in one MMC monotherapy strain and all MMC + Rif strains, with two strains sharing the same mutation in Alanine 248 in *uvrB* (Fig. 4). Furthermore, three of the six evolved strains contained mutations in the gene of an FMN-dependent NADH-azoreductase, *azo1*. Two strains were mutated in the same position (Alanine 94) and the third strain in a nearby position (Serine 86). As the mutations in Ala 94 in *azo1* and the Ala248Val mutation in *uvrB* both were found in several independently evolved strains, it is highly likely that they are the reason for the increased mitomycin C MIC.

To further investigate the importance of the NER system and Azo1 in mitomycin C susceptibility, we wished to use knock-out mutants of the relevant genes. The commercially available Nebraska Transposon Mutant Library consists of ~2,000 knock-out mutants in the methicillin-resistant *S. aureus* USA300 FPR3757 background generated using transposon mutagenesis (64). We identified four transposon mutants with disrupted *uvrA*, *uvrB*, *uvrC,* or *azo1* genes from the library. The Δ*uvrA*, Δ*uvrB,* and Δ*uvrC* mutants all lack the NER system since each of the three gene products are essential for the repair system to perform its function (65). We hypothesized that if the NER system or Azo1 are involved in mitomycin C susceptibility, we would see a difference in growth inhibition and cell death from the wild type to the knock-out mutants.

Δ*uvrA*, Δ*uvrB,* and Δ*uvrC* mutants were significantly more susceptible to mitomycin C than the wild type with MIC values of 0.0625 µg/mL and minimum biocidal concentration (MBC) values of 0.125 µg/mL for all three knock-out mutants compared with an MIC of 0.25 µg/mL and an MBC of 0.25 µg/mL for the wild type (Table 6). This finding underlines that the NER system plays a key role in the survival of *S. aureus* following exposure to mitomycin C. Furthermore, the Δ*azo1* mutant was less susceptible to mitomycin C with an MIC of 0.5 µg/mL and MBC of 1 µg/mL (Table 6). This finding suggests that Azo1 might play a role in the activation, transport, or proper function of mitomycin C.

**TABLE 6:** Minimum inhibitory concentrations and minimum biocidal concentrations of mitomycin C against knock-out mutants^*a*^

Strain	MIC (µg/mL)	MBC (µg/mL)
Wild type	0.25	0.25
Δ*uvrA*	0.0625	0.125
Δ*uvrB*	0.0625	0.125
Δ*uvrC*	0.0625	0.125
Δ*azo1*	0.5	1

^*a*^ MIC and MBC values were assessed in TSB against methicillin-resistant *S. aureus* USA300 FPR3757 WT and ΔuvrA, ΔuvrB, ΔuvrA, and Δazo1 of the same stain.

The difference in susceptibility of the knock-out mutants taken together with the observation of point mutants in the *uvrB, uvrC,* and *azo1* genes all suggest that the NER system and Azo1 play a role in mitomycin C susceptibility. However, these genes have not previously been associated with resistance to mitomycin C, and further experiments are needed to verify the connection between the corresponding proteins and the potential for developing resistance to mitomycin C.

## DISCUSSION

In this study, we showed that resistance to rifampicin, ciprofloxacin, vancomycin, and daptomycin occurs within days or weeks during adaptive laboratory evolution, while mitomycin C was less prone to resistance since the MIC only increased by twofold during mitomycin C monotherapy and mitomycin C + rifampicin combination therapy.

Adaptive evolution under mitomycin C exposure revealed new insights into how bacteria can protect themselves from this drug. The elevated MIC for mitomycin C was most likely linked to mutations in *azo1*, *uvrB*, and *uvrC*. During NER, UvrABC recognises damaged DNA, cleaves the phosphodiester bond, and subsequently removes 10–15 bp of the damaged DNA which can then be filled out by DNA polymerase I (65). UvrABC has further been implicated in the reparation of mono- and interstrand crosslinks following mitomycin C treatment (66). In line with this, we found that NER-deficient mutants were significantly more susceptible to mitomycin C than the wild type (Table 6). Therefore, it is highly likely that the mutations we identified in *uvrB* and *uvrC* caused the increase in mitomycin C MIC seen here.

The three mutations observed in *uvrB* in strains MMC 2, MMC + Rif 2, and MMC + Rif 3 are all located in domain 2 of the gene (residues 154–251). In a study from 2004, Truglio et al. found that domain 2 is essential for the catalytic activity of UvrB during both incision and strand separation and that the domain is essential during recruitment of UvrB to UvrA for proper NER function (67). We observed an Arg194Gln mutation in the strain MMC 2 leading to an exchange of a positively charged amino acid with an uncharged amino acid. Truglio et al. found that the residue Arg 194 is very highly conserved across different species suggesting that the residue plays a fundamental role in the functionality of the NER. Finally, the findings by Truglio et al. underlined the suggestion that the recruitment of UvrB to UvrA is the rate-limiting step in UvrABC-mediated excision repair. We hypothesize that the mutations we identified in domain 2 of UvrB improve the recruitment of UvrB to UvrA, thereby leading to a more efficient NER system that may repair the damages by mitomycin C. We further identified a single *uvrC* mutation in all mitomycin C-treated strains. This Glu197Lys mutation is located in the four-helix bundle of UvrC, which is closely located in the UvrB-binding domain (68). It is likely that the mutation causes a change in the electrostatic interactions between UvrB and UvrC since the net charge is changed by two units at the mutated residue. However, this hypothesis would need to be tested in a UvrB-UvrC interaction assay.

Our study identified for the first time the FMN-dependent NADH-azoreductase, *azo1*, as a gene of interest in decreased susceptibility to mitomycin C. Azo1 was first described in 2005 (69), but there are limited reports on the involvement of the enzyme in antibiotic resistance in *S. aureus*. However, loss-of-function caused resistance against the quinolone JSF-3151 (70) in line with our finding of decreased mitomycin C susceptibility in a Δ*azo1* mutant. Furthermore, mutations in an Azo1-homologue, AzoR, in *E. coli* conferred resistance to thiol-specific stress from electrophilic quinones and were shown to reduce multiple quinone compounds resembling mitomycin C (71). Upon entering the cell, the quinone ring of mitomycin C needs to be reduced in order for the drug to be activated (72). This is most commonly obtained enzymatically, but chemical reductants may also activate the drug. Multiple eukaryotic bioreductive enzymes capable of mitomycin C bioactivation have been identified (72) due to the long history of using mitomycin C in bladder cancer. However, the use of mitomycin C against pathogens is more recent, and the literature on reducing enzymes capable of mitomycin C bioreduction in prokaryotes is limited. We hypothesize that Azo1 is capable of activating mitomycin C, thereby leading to the drug being able to exert its full efficacy. Azo1 would not be the only enzyme in *S. aureus* with this ability since the Δ*azo1* mutant was only 2-fold less susceptible than the wild type, and we would expect a larger difference in susceptibility between the wild type and the Δ*azo1* mutant. This is the first report of mutations in *azo1* correlating with decreased susceptibility to mitomycin C, and further studies are needed to elucidate if Azo1 is capable of enzymatic activation of mitomycin C.

We also gained new insights into the evolution of daptomycin resistance, namely that several different mechanisms seem to contribute simultaneously to raise MIC, and that the high MIC also correlates with slower growth rates. Daptomycin is a positively charged lipopeptide drug, and the proposed mechanism of action is through calcium-dependent insertion into the bacterial membrane with subsequent oligomerization leading to membrane disruption (73). Therefore, one can expect that daptomycin resistance may occur due to changes in the membrane charge or fluidity. Jones et al. demonstrated increased membrane fluidity and net positive surface charge of daptomycin-resistant isolates as well as increased translocation of the positively charged lysyl phosphatidylglycerol (LPG) to the membrane in *S. aureus* (74). LPG is synthesized by the bifunctional membrane protein MprF, which further has flippase activity (51). Here, we found that all evolved daptomycin-resistant strains had mutations in the *mprF* gene (Fig. 4), and it seems most likely that daptomycin resistance occurs due to mutations in *mprF* that cause increased synthesis of LPG, which in turn leads to a more fluid and positively charged membrane that interacts poorly with daptomycin (75).

Evolved strains Dapto strain 1, Dapto strain 2, and Dapto + Rif strain 1 had substantially higher MIC values than the other evolved strains, and they all displayed slower growth rates (Table 1; Fig. 3A). Dapto 1 and Dapto 2 had similar mutations in the *mprF* gene with mutations in leucine 826, and Dapto + Rif 1 had a truncated *mprF* with a Pro314Leu mutation. A previous study generated point mutations in *mprF* in a clinical MRSA strain to achieve daptomycin resistance, and these mutations also caused a decrease in growth rate (76). Although there is a connection between mutation of leucine 826 and slow growth for Dapto strains 1 and 2, Dapto + Rif strain 3 had a similar mutation at leucine 826 and did not display slow growth or a high MIC. Therefore, mutation at this amino acid alone is not responsible for high daptomycin resistance or slow growth. The mutations responsible for slowing the growth rate therefore remain to be identified, and our result underlines the complex mechanisms for daptomycin resistance, which relies on several mutations.

The evolution of rifampicin resistance during combination therapy also revealed insights into what drives rifampicin resistance when other antibiotics are used simultaneously. It appears that high rifampicin resistance can evolve even at very low concentrations of rifampicin because a single point mutation can increase MIC values by >100-fold. This was evident in Dapto + Rif 1, which never received more than 8 µg/mL rifampicin during the study, but subsequently had an MIC of 512 µg/mL (Table 1) conferred by a single mutation in *rpoB* (Ser487Leu). To the best of our knowledge, this mutation has not previously been reported in rifampicin-resistant *S. aureus*. In contrast, a different mutation in *rpoB* at a nearby location (Ala477Val) in Dapto + Rif strain 3 only caused a moderate (twofold) increase in MIC.

In general, combination therapy did not prevent the emergence of rifampicin resistance for the antibiotics tested in this study. It was therefore remarkable that strains evolved under mitomycin C and rifampicin combination therapy did not develop resistance to rifampicin. The maximum rifampicin concentration these strains were exposed to was 0.008 µg/mL, and it was confirmed in a subsequent experiment that this concentration is sufficient to drive rifampicin resistance. The lack of resistance in samples evolved under rifampicin and mitomycin C combination therapy must therefore be ascribed to the presence of mitomycin C, possibly due to the inhibition of spontaneous mutations by this drug (32). This finding was further underlined by the lack of *rpoB* mutations commonly associated with rifampicin resistance in two out of three strains. While this is an encouraging result, we must note that the use of rifampicin in the clinic is at higher concentrations, even when used in combination therapy. We can therefore not rule out that rifampicin resistance develops under those conditions, even if rifampicin is combined with mitomycin C. Further research should therefore investigate more deeply the “protective” effect of mitomycin C in relation to rifampicin resistance.

In summary, resistance to rifampicin and the primary antibiotic did develop during combination therapy; however, it was delayed compared to monotherapy. We observed similar genetic mutations for strains receiving combinations as compared with monotherapy, for example, mutations in *azo1* (mitomycin C resistance), *parC/grlA* and *perE/grlB* (ciprofloxacin resistance), *rpoB* (rifampicin resistance), and mutations in genes involved in cell wall biosynthesis (vancomycin and daptomycin resistance). Therefore, combination therapy did not affect the mutation targets, and we expect that any strategies to avoid resistance development during monotherapy would further be effective in avoiding resistance development during combination therapy.

## MATERIALS AND METHODS

### Strains, growth conditions, and antibiotics

*S. aureus* ATCC 29213 WT was used to generate evolved antibiotic-resistant strains during adaptive laboratory evolution. *S. aureus* was routinely grown overnight in tryptic soy broth (TSB, T8907, Sigma Aldrich) in Erlenmeyer flasks and incubated at 37°C, 180 rpm unless otherwise stated. Antibiotics used for adaptive laboratory evolution and determination of the MIC were mitomycin C (J63193.MA, Thermo Scientific), vancomycin (Bactocin, MIP Pharma GmbH), ciprofloxacin (17850, Sigma Aldrich), daptomycin (Cubicin, Merck Sharp & Dohme), and rifampicin (Rifadin, Sanofi S.r.I.).

### MIC determination

MICs were determined using broth dilution in TSB, but otherwise following the ISO standard (77). Briefly, *S. aureus* was diluted to a turbidity of 0.05 and inoculated in twofold antibiotic dilution series in TSB in 96-well plates, yielding a bacterial concentration of 5 × 10^5^ CFU/mL. Plates were incubated overnight at 37°C, 50 rpm, and optical density at 600 nm (OD_600_) was measured to detect growth. MIC was determined as the lowest antibiotic concentration with OD_600_ <20% of the growth control. 50 µg/mL Ca^2+^ was added in samples with daptomycin.

### Adaptive laboratory evolution assay

Three independent colonies (parents 1, 2, and 3) were inoculated in TSB and incubated overnight. The three samples were diluted to a turbidity of 0.05 and inoculated in antibiotic dilution series in 96-well plates. The antibiotic treatments included mitomycin C, vancomycin, ciprofloxacin, daptomycin, rifampicin, mitomycin C + rifampicin, vancomycin + rifampicin, ciprofloxacin + rifampicin, and daptomycin + rifampicin. 96-well plates were incubated overnight at 37°C and 50 rpm, and subsequently, the MIC was determined by reading the plates in a plate reader at 600 nm. Bacteria from the dilution step immediately below MIC were transferred to a fresh dilution series of antibiotic treatments and incubated overnight. The range of antibiotic concentrations used was from 0.25- to 2-fold the MIC value from the previous day. This cycle was repeated 42 times. As the MIC increased, bacteria surviving higher antibiotic concentrations were transferred to the next cycle with increased antibiotic concentrations, thereby adapting the assay to the development of resistance. After 42 cycles, evolved strains were streaked on TSB agar and grown overnight without antibiotics. Subsequently, a single colony was picked for each strain for storage at −80°C in 25% glycerol. Freezer cultures were streaked onto TSB agar for downstream experiments and for extracting DNA for genome sequencing. Evolved strains were named after the treatment they received during adaptive laboratory evolution, that is, MMC, Vanco, Cipro, Dapto, Rif, MMC + Rif, Vanco + Rif, Cipro + Rif, and Dapto + Rif. In combination treatments, the relative concentration of rifampicin to the primary antibiotic was held constant throughout the adaptive evolution. For each, three independently evolved strains were generated, yielding 27 uniquely evolved strains.

To test if low concentrations of rifampicin could lead to rifampicin resistance, we additionally performed a 7-day evolution experiment. Here, the evolution experiment was set up similarly to what is described above, but in twofold rifampicin dilution series with concentrations up to 0.008 µg/mL. Mitomycin C was added at 0, 0.25, or 5 × MIC to the dilution series. After overnight growth, MIC was determined, and bacteria from the highest rifampicin concentration where there was visible growth were transferred to a fresh dilution series. This cycle was repeated for 7 days and subsequently, samples were inoculated into an Erlenmeyer flask with fresh TSB without antibiotics and grown overnight.

### Growth curves

Overnight cultures of parent and evolved strains were diluted 1,000-fold in TSB and incubated in duplicate in a 96-well plate in a shaking plate reader at 37°C, where plates were shaken for 10 s at 200 rpm immediately before each measurement. OD_600_ was measured every 30 min for 24 h.

### Biofilm formation

Overnight cultures of parent and evolved strains were diluted to OD_600_ = 1 in TSB and added to 96-well plates with peg lids pre-conditioned with TSB. After 30 min attachment, peg lids were transferred to fresh TSB, and 96-well plates were incubated at 37°C, 50 rpm for 3 days with exchange of media every 24 h. Subsequently, peg lids were washed twice by transferring to 1× M9 salts (M6030, Sigma Aldrich) for 30 s each. The peg lids were sonicated in M9 salts for 10 min to detach biofilms, and CFU enumeration was subsequently performed on the sonicate.

### Genome sequencing, assembly, and bioinformatics analyses

DNA was extracted from overnight cultures using the DNeasy UltraClean Microbial Kit following the manufacturer’s protocol (QIAGEN, 12224-50) and prepared for sequencing using the Nextera XT DNA Library Prep Kit (Illumina, FC-131-1024) to tagment the DNA with adapter sequences. The DNA was amplified, and adapter and index sequences were added through PCR. AMPure XP magnetic beads were used for DNA purification, removal of primers, and short fragments. Finally, the sequences were sequenced on an Illumina MiSeq Next Generation sequencer.

Sequenced genomes were quality controlled using FastQC (78) and trimmed based on per base sequence content using Trimmomatic (79). Genomes were assembled and mapped from a reference genome (NCBI: *S. aureus* ATCC 29213, assembly GCA_001267715.2) using BactSNP (80). Assemblies were quality controlled using Quast (81) and SNP annotation was performed using SnpEff (82). In order to validate the located SNPs, genomes were further annotated with Prokka (83), and searches for specific mutations were conducted using BLASTp (84).

### Statistical analyses

Ordinary one-way ANOVA was used for bar graphs with a *post hoc* uncorrected Fisher’s test. Absolute values of the MIC are shown for a minimum of three replicates. GraphPad Prism was used for all statistical analyses (v.9.5.1 (733) for Windows, GraphPad Software, San Diego, CA, USA, www.graphpad.com).

**TABLE 7:** Accession numbers for sequenced genomes

Sample name	Accession number	URL
Parent 1	SAMN46357333	https://www.ncbi.nlm.nih.gov/biosample/46357333
Parent 2	SAMN46357334	https://www.ncbi.nlm.nih.gov/biosample/46357334
Parent 3	SAMN46357335	https://www.ncbi.nlm.nih.gov/biosample/46357335
MMC 1	SAMN46357336	https://www.ncbi.nlm.nih.gov/biosample/46357336
MMC 2	SAMN46357337	https://www.ncbi.nlm.nih.gov/biosample/46357337
MMC 3	SAMN46357338	https://www.ncbi.nlm.nih.gov/biosample/46357338
Vanco 1	SAMN46357339	https://www.ncbi.nlm.nih.gov/biosample/46357339
Vanco 2	SAMN46357340	https://www.ncbi.nlm.nih.gov/biosample/46357340
Vanco 3	SAMN46357341	https://www.ncbi.nlm.nih.gov/biosample/46357341
Cipro 1	SAMN46357342	https://www.ncbi.nlm.nih.gov/biosample/46357342
Cipro 2	SAMN46357343	https://www.ncbi.nlm.nih.gov/biosample/46357343
Cipro 3	SAMN46357344	https://www.ncbi.nlm.nih.gov/biosample/46357344
Dapto 1	SAMN46357345	https://www.ncbi.nlm.nih.gov/biosample/46357345
Dapto 2	SAMN46357346	https://www.ncbi.nlm.nih.gov/biosample/46357346
Dapto 3	SAMN46357347	https://www.ncbi.nlm.nih.gov/biosample/46357347
Rif 1	SAMN46357348	https://www.ncbi.nlm.nih.gov/biosample/46357348
Rif 2	SAMN46357349	https://www.ncbi.nlm.nih.gov/biosample/46357349
Rif 3	SAMN46357350	https://www.ncbi.nlm.nih.gov/biosample/46357350
MMC + Rif 1	SAMN46357351	https://www.ncbi.nlm.nih.gov/biosample/46357351
MMC + Rif 2	SAMN46357352	https://www.ncbi.nlm.nih.gov/biosample/46357352
MMC + Rif 3	SAMN46357353	https://www.ncbi.nlm.nih.gov/biosample/46357353
Vanco + Rif 1	SAMN46357354	https://www.ncbi.nlm.nih.gov/biosample/46357354
Vanco + Rif 2	SAMN46357355	https://www.ncbi.nlm.nih.gov/biosample/46357355
Vanco + Rif 3	SAMN46357356	https://www.ncbi.nlm.nih.gov/biosample/46357356
Cipro + Rif 1	SAMN46357357	https://www.ncbi.nlm.nih.gov/biosample/46357357
Cipro + Rif 2	SAMN46357358	https://www.ncbi.nlm.nih.gov/biosample/46357358
Cipro + Rif 3	SAMN46357359	https://www.ncbi.nlm.nih.gov/biosample/46357359
Dapto + Rif 1	SAMN46357360	https://www.ncbi.nlm.nih.gov/biosample/46357360
Dapto + Rif 2	SAMN46357361	https://www.ncbi.nlm.nih.gov/biosample/46357361
Dapto + Rif 3	SAMN46357362	https://www.ncbi.nlm.nih.gov/biosample/46357362

## Data Availability

Assembled genomes are available in NCBI under the BioProject ID PRJNA1214057. The accession number and URL for each individual genome are provided in Table 7.

## SUPPLEMENTAL MATERIAL

Supplemental material aac.01483-24-s0001.docx Tables S1 and S2; Fig. S1.

ASM does not own the copyrights to Supplemental Material that may be linked to, or accessed through, an article. The authors have granted ASM a non-exclusive, world-wide license to publish the Supplemental Material files. Please contact the corresponding author directly for reuse.
