# Supplementary material for: Combination therapy delays antimicrobial resistance after adaptive laboratory evolution of Staphylococcus aureus
Source: Antimicrob Agents Chemother. 2025 Mar 14;69(4):e01483-24. doi: 10.1128/aac.01483-24 (PMC11963546; doi:10.1128/aac.01483-24)
Supplement: Supplemental material — Tables S1 and S2; Fig. S1. [file aac.01483-24-s0001.docx]

Supplementary materials to: Combination therapy delays antimicrobial resistance after adaptive laboratory evolution of *Staphylococcus aureus*

By authors: Maiken Engelbrecht Petersen, Amanda Batoul Khamas, Lars Jørgen Østergaard and Rikke Louise Meyer

**Table S1: Additional SNPs located in the genomes from evolved strain post-evolution**. These SNPs were either not repeated across replicates or they were not expected relevant. *Gene products were truncated in the N-terminal with the position indicated being the new start codon. **Missing start codon. Codon positions are S. aureus relative

| Strain | Additional genetic mutations |
| --- | --- |
| MMC 1 | Protein-(glutamine-N5) methyltransferase Ala208Pro  *rnase Y* Ala154Thr  YggS family pyroxidal phosphate enzyme Ala126Pro  Histidine kinase Glu358STOP  *purR* Arg5Gln  transcription-repair coupling factor Asp768Tyr  *addA* Arg324Thr  *comK* Asn116Tyr |
| MMC 2 | phosphotransferase Ala43Pro  hypothetical protein Ser325Leu  *fmtA* Gly90Arg  hypothetical protein Lys253STOP  *sodA* Glu54STOP  glycerophosphodiester phosphodiesterase Leu405Phe  *mobA* Arg132Leu  sodium:alanine symporter Arg514Cys  transcriptional antiterminator Glu560Val |
| MMC 3 | *mprF* His540Arg  *tcaA* Gln79STOP  luciferase-like monooxygenase Ala195Val |
| MMC+Rif 1 | peptide deformylase Leu90Val  histidine-tRNA ligase Glu177Lys  *mgrA* Ala110Asp  glutamate-tRNA ligase Glu428Asp  *nfsA* Asp204His  chorismate mutase Pro47Leu  acetyl-CoA acetyltransferase Gly383Ala  *saeS* Thr307Lys  hypothetical protein AKN43_009005 Glu127Asp |
| MMC+Rif 2 | *ftsW* Leu361Phe  trehalose permease IIC protein Ile11Met  *ebh* Leu9783Val  phosphotransferase Gln26fMet*  hypothetical protein AKN43_013075 Arg99Pro |
| MMC+Rif 3 | *plsY* Ser161Leu  *ftsW* Leu361Phe  trehalose permease IIC protein Ile11Met  *ebh* Leu9783Val  phosphotransferase Gln26fMet*  hypothetical protein AKN43_013075 Arg99Pro |
| Vanco 1 | Thioredoxin reductase Ala140Val  RNA-binding transcriptional accessory protein Thr338Ile |
| Vanco 2 | hypothetical protein AKN43_005665 Gly128Asp |
| Vanco 3 | ABC transporter ATP-binding protein Glu69Gly  adenine phosphoribosyltransferase Pro65Leu |
| Vanco+Rif 1 | *fmtA* Ala285Glu  *ssaA* STOP lost |
| Vanco+Rif 2 | *fmtA* Ala285Glu  *icaB* Tyr132Phe |
| Vanco+Rif 3 | Phosphotransferase Gln26fMet*  hypothetical protein AKN43_013075 Arg99Pro  *ftsW* Leu361Phe  trehalose permease IIC Ile11Met  *plsY* Ser161Leu  *uvrB* Ala248Val  *ebh* Leu9783Val |
| Cipro 1 | *tilS* Gly429Arg |
| Cipro 2 | *nrdI* Ala85Gly |
| Cipro 3 | *gdpP* Ala626Val |
| Cipro+Rif 1 | - |
| Cipro+Rif 2 | - |
| Cipro+Rif 3 | *comEC/rec2* Pro232Ala |
| Dapto 1 | *aroB* Gly98Val  *apt* Gly124Val  hypothetical protein AKN43_011940 Gly22Glu |
| Dapto 2 | *rpsU* Glu10Lys  *gpsA* Gln51STOP  Metal-dependent hydrolase Trp311Cys |
| Dapto 3 | *fabH* Pro273Ser  *addB* Asp339Glu  *dtlD* Gly366Asp  Na^+^/H^+^ antiporter subunit D Gly97STOP |
| Dapto+Rif 1 | *DDl* Cys7Ser  Metal-dependent hydrolase Met1?** |
| Dapto+Rif 2 | - |
| Dapto+Rif 3 | *cosEA* His288Gln  *tpx* Asp150Gly |
| Rif 1 | - |
| Rif 2 | *purR* Val30Leu |
| Rif 3 | - |


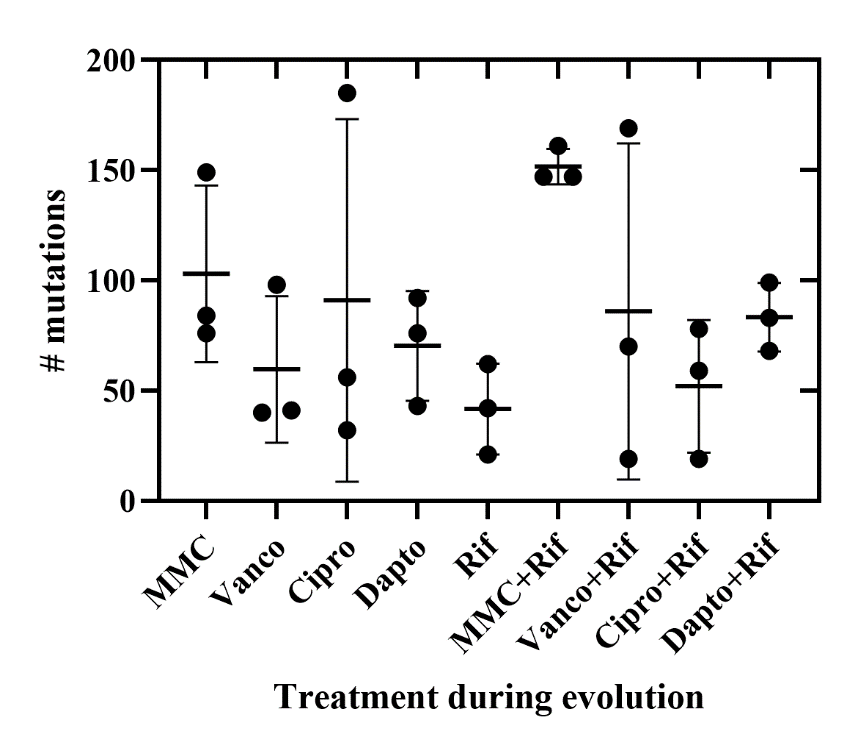

**Figure 1. Total number of single nucleotide polymorphisms in evolved S. aureus.** SNPs were identified using BactSNP. SNPs were identified for all strains including the parent colonies pre-evolution. The SNPs identified from the parent colonies were subtracted from the respective evolved strains. The total number of mutations include missense mutations, synonymous mutations, stop gained, stop lost, start lost, mutations upstream of genes, mutations downstream of genes and other intergenic mutations.

**Table S2. Distribution of mutations in evolved S. aureus** Missense: Genetic mutations causing an amino acids change. Synonymous: Gentic mutations causing no change in amino acids. Stop gained: Genetic mutation leading to premature stop codon. Stop lost: The stop codon has been mutated to no longer be present. Start lost: The start codon ATG has mutated. Upstream: Mutation positioned upstream (within 5 kb) of the most distal transcription start site. Downstream: Mutation positioned downstream (within 5 kb) of the most distal poly(A) addition site. Intergenic: Mutations located before a transcription start site and after the poly(A) addition site, but not including upstream or downstream mutations.

| Strain | Mis-sense | Synonymous | Stop gained | Stop lost | Start lost | Up-stream | Down-stream | Inter-genic |
| --- | --- | --- | --- | --- | --- | --- | --- | --- |
| MMC 1 | 8 | 0 | 1 | 0 | 0 | 27 | 40 | 8 |
| MMC 2 | 8 | 0 | 2 | 0 | 0 | 47 | 76 | 16 |
| MMC 3 | 4 | 1 | 1 | 0 | 0 | 28 | 32 | 10 |
| Vanco 1 | 5 | 0 | 0 | 0 | 0 | 9 | 18 | 8 |
| Vanco 2 | 4 | 1 | 0 | 0 | 0 | 40 | 35 | 18 |
| Vanco 3 | 5 | 0 | 0 | 0 | 0 | 8 | 10 | 18 |
| Cipro 1 | 3 | 1 | 0 | 0 | 0 | 83 | 92 | 6 |
| Cipro 2 | 4 | 0 | 0 | 0 | 0 | 24 | 20 | 8 |
| Cipro 2 | 4 | 0 | 0 | 0 | 0 | 7 | 11 | 10 |
| Dapto 1 | 6 | 0 | 0 | 0 | 0 | 10 | 19 | 8 |
| Dapto 2 | 5 | 0 | 1 | 0 | 0 | 36 | 38 | 12 |
| Dapto 3 | 7 | 1 | 1 | 0 | 0 | 30 | 29 | 8 |
| Rif 1 | 3 | 0 | 0 | 0 | 0 | 5 | 5 | 8 |
| Rif 2 | 2 | 1 | 1 | 0 | 0 | 20 | 30 | 8 |
| Rif 3 | 2 | 1 | 0 | 0 | 0 | 10 | 13 | 16 |
| MMC+Rif 1 | 12 | 0 | 0 | 0 | 0 | 43 | 78 | 14 |
| MMC+Rif 2 | 7 | 0 | 0 | 0 | 0 | 59 | 73 | 22 |
| MMC+Rif 3 | 8 | 0 | 0 | 0 | 0 | 46 | 67 | 26 |
| Vanco+Rif 1 | 3 | 0 | 0 | 1 | 0 | 2 | 5 | 8 |
| Vanco+Rif 2 | 5 | 0 | 1 | 0 | 0 | 34 | 22 | 8 |
| Vanco+Rif 3 | 8 | 0 | 0 | 0 | 0 | 56 | 75 | 30 |
| Cipro+Rif 1 | 2 | 0 | 0 | 0 | 0 | 6 | 1 | 10 |
| Cipro+Rif 2 | 1 | 0 | 0 | 0 | 0 | 27 | 15 | 16 |
| Cipro+Rif 3 | 3 | 1 | 0 | 0 | 0 | 19 | 31 | 24 |
| Dapto+Rif 1 | 5 | 0 | 2 | 0 | 1 | 22 | 30 | 8 |
| Dapto+Rif 2 | 5 | 1 | 0 | 0 | 0 | 30 | 35 | 12 |
| Dapto+Rif 3 | 7 | 1 | 0 | 0 | 0 | 33 | 40 | 18 |
